# Supplementary material for: Physics of martial arts: Incorporation of angular momentum to model body motion and strikes
Source: PLoS One. 2021 Aug 10;16(8):e0255670. doi: 10.1371/journal.pone.0255670 (PMC8354461; doi:10.1371/journal.pone.0255670)
Supplement: Attachment — Submitted filename: renamed_67bcf.pdf [file pone.0255670.s007.pdf]

Authors' reply to reviewer comments for manuscript PONE-D-21-04861.

Dear Dr. Hadizadeh,

We thank the reviewers and the Journal editorial team for their helpful and insightful comments. In response, we have made the following changes to our manuscript and hope that, with these changes, our manuscript will be suitable for publication.

One area of substantial re-write was more carefully explaining how the three collision models presented in the manuscript apply to material arts movements. Given that martial arts are typically practiced by standing on solid ground, it was not obvious why we would offer a model involving a movable platform. We now provide (we hope) a more full motivation for developing these model collisions and why the obtained results can be applied to martial arts movements.

In order, our responses (in **boldface**) to specific comments are:

[From Journal editorial team]

1) "Please ensure that your manuscript meets PLOS ONE's style requirements […]"

We have check and made corrections to the formatting using the style guidelines.

2) "In the manuscript, please clarify how […]"

The person in the videos and images are of one author's daughter. The videos were acquired with her permission (and to be honest, some measure of glee at appearing in a scientific Journal). We have completed the PLOS consent form and included the requested information within the manuscript.

3) "Please ensure that you refer to Figure 2-7 in your text […]"

We double-checked that the Figures are numbered correctly and are matched to the (numbered) figure captions.

4) "We note the Figures 3,4,5 and supporting information videos […]"

As noted above, the completed PLOS consent form has been attached, and the requested verbiage has been added in the Methods section.

[Reviewer #1]

1) "In Case 3 the person is jumping tangentially […]"

We have discovered that this case is very similar to an example problem discussed in Taylor's "Classical Mechanics", as Example, 3.4. To quickly answer the reviewer's questions, once the person has jumped off the disk, there is no longer an exerted force or torque acting on the disc, so it will rotate about its own center of mass and not the system center of mass. We have added some clarifying discussion in the manuscript in this section.

2) When discussing the equations for this system, l is listed as an unknown, but l can be calculated in terms of m, M, and R. Should this relationship be listed with Eqs 2, 3 and 4?

The reviewer is correct, we apologize for the error and have corrected this and explicitly wrote the expression for 'l' that is used to solve the three conservation equations.

3) In the paragraph starting with line 125 the authors states that the two equations above should hold for a martial artist […]

We apologize for the confusion, the text has been removed as it was confusing.

4) The paragraph at line 149 notes […]

We re-wrote this paragraph to more clearly state that in our model, the transfer of energy is more important than the transfer of momentum in terms of damage to a target.

5) As a tiny detail, E_i and E_f in line 108 should probably be E_initial and E_final for consistency.

This has been corrected.

6) The description found beginning at line 49 and the Figure 1 itself are a bit confusing. […]

We (authors) have thought carefully about this section (and figure), as we agree that the discussion was somewhat disjoint from the main subject of our manuscript. However, we believe that it remains useful from a pedagogical perspective and have cleaned up the Figure in accordance with reviewer's suggestions. We have also edited the section to bring more clarity and relevance to this example.

7) Figure 2 could probably be fixed up a little as well by placing the tail of the R vector at the visual center of the circle. If the object little m is truly starting at

a distance R from the center. It would probably be good to place the object much closer to the rim of the circle.

We have made the suggested changes to Figure 2 to improve 'readability'.

8) In general the paper presents an interesting analysis of martial arts using angular momentum at two different levels of complexity. The paper could benefit from an extra pass of copy-editing, some work to clarify and clean up a few of the figures, and a bit of work to make sure that text provides a little extra clarification on a few of the statements. My biggest concern is finding confirmation that Case 3 is properly being modeled regarding the use of the center of mass for the moment arm of the person and inconsistencies that might exist between that and the unconstrained disk.

We appreciate the kind words and have spent time copy-editing the text. We have thoroughly checked our results of these model collision problems and are confident the results are correct. The authors agree that Case 3 is indeed an interesting model problem and were excited to go through the process of solving it.

[Reviewer #2]

1) Overall this document is the beginning of some good work[…]

We appreciate the summary and have eliminated vague, off-topic statements , and extraneous information throughout

2) 2) To start, the title "Physics of Martial Arts" is far too broad […]

We have modified the title to provide more specific goals of the manuscript.

3) The abstract is vague and non-specific, at least at the beginning. […]

We have modified the abstract to better convey the intention of our manuscript.

4) I do not see how the entire section "Linear and angular momentum of rectilinear motion" as expressed has anything to do with the rest of the paper. I do not see how Figure 1 is relevant to later sections.

As mentioned above to Reviewer #1, We (authors) have thought carefully about this section (and figure), as we agree that the discussion was somewhat disjoint from the main subject of our manuscript. However, we

believe that it remains useful from a pedagogical perspective and we have also edited the section to bring more clarity and relevance to this example.

5) Concerning Case 3 in the section […]

We agree that Case 3 offers many opportunities for conceptual mistakes- one of the primary reasons we have included this in our manuscript. To answer the primary question "Does the disk rotate about its own center of mass or the center of mass of the system?", we note that after the person has jumped, subsequent motion of the disk (and person) evolves in a force- and torque-free manner. This is unlike two orbiting bodies that rotate about the system center of mass, because in orbital motion there is a force exerted between the two objects (gravity, for example). Taylor's "Classical Mechanics" has a worked example (Example 3.) that analyses a similar problem in terms of forces and torques, and we now reference this in our manuscript.

6) I prime in Equation 4 is not previously defined. […]

We apologize for the error, I' should have been I_d. We have corrected this typographical error.

7) You might overtly state location equation of the center of mass l. It would make the development clearer.

We have added this expression, it can be found just above equation 2.

8) Equation 5 appears to be missing a negative sign.

We thank the reviewer for catching this typographical error, we corrected the sign in equations 5, 7, 9, and 11.

9) What is the purpose of the "0.1" in front of […]

We apologize, that was a formatting error and has been removed.

10) Concerning line 130, it is not clear to me, […]

We have re-written that section to bring more clarity in how our model collision problems relate to martial arts movements.

11) The analysis of the three kicks was interesting, but I felt the conclusions were weak and non-specific. The motion was described in physical terms, but I'm not sure what was to be concluded from these descriptions.

At this time, the revised analysis and conclusions are all we are prepared to state; this summer we hope to obtain high-speed video of these kicks and perform a quantitative analysis to form the nucleus of a follow-on report.

12) The moment of inertia tensor model was even better, […]

We appreciate the reviewer checking our results- the reviewer is quite correct, we made several mistakes we have since corrected. Going through this comment in detail: We corrected the moment of inertia tensor (equation 15) and Figure 7. Next, we carefully considered the common center of rotation for all three cylinders and estimated it to be the common point of intersection of all three cylinder axes. This estimate places the center of rotation on the axis of the trunk cylinder a distance 'R' from the bottom face. This location has been indicated in Figure 6 with a note added in the Figure 6 caption. Regarding obtaining additional conclusions, while it could be interesting to for example, perform a comparative analysis of a roundhouse kick and an axe kick, at this time we are not prepared to expand our results.

In conclusion, we believe we have adequately responded to all reviewer comments and suggestions, and have edited our manuscript to conform with Journal requirements. We appreciate this opportunity to improve our manuscript.

Sincerely,

Andrew Resnick
