# Supplementary material for: Physics of martial arts: Incorporation of angular momentum to model body motion and strikes
Source: PLoS One. 2021 Aug 10;16(8):e0255670. doi: 10.1371/journal.pone.0255670 (PMC8354461; doi:10.1371/journal.pone.0255670)
Supplement: Attachment — Submitted filename: renamed_3096a.pdf [file pone.0255670.s008.pdf]

Authors' second reply to additional reviewer comments for manuscript PONE-D-21-04861.

Dear Dr. Hadizadeh,

We thank the reviewers and the Journal editorial team for their helpful and insightful comments. In response, we have made the following changes to our manuscript and hope that, with these changes, our manuscript will be suitable for publication.

One reviewer correctly noted the following typographical errors:

- Line 15: "a action-reaction" should be "an action-reaction"
- Line 63: I think you mean to say the y-intercept is ... not the x-intercept is (the x-intercept x0)
- Line 96: You might want to consider a new sentence with "For example, ..."
- Line 108 and beyond: I did not catch the inconsistent use of capitalization with "Case 1", "case 1". […]
- Line 256: a figure reference is missing.

All of these errors have been corrected and Figure #1 has also been corrected, adding 'x_0' as per the reviewer request.

In conclusion, we believe we have adequately responded to all reviewer comments and suggestions, and have edited our manuscript to conform with Journal requirements. We appreciate this opportunity to improve our manuscript.

Sincerely,

Andrew Resnick
